# What happened among Japanese children from school closure due to COVID-19 after school re-opening? Changes in sleep habits and dietary intake

**DOI:** 10.1017/jns.2022.116

**Published:** 2023-01-23

**Authors:** Minami Sugimoto, Kentaro Murakami, Satoshi Sasaki

**Affiliations:** 1 Institute for Future Initiatives, University of Tokyo, Tokyo, Japan; 2 Department of Environmental and Occupational Health, School of Medicine, Toho University, Tokyo, Japan; 3 Department of Social and Preventive Epidemiology, School of Public Health, University of Tokyo, 7-3-1 Hongo, Bunkyo-ku, Tokyo 113 0033, Japan

**Keywords:** Dietary intakes, Latent class analysis, School-aged children, Sleep habits, Temporal sleep and eating patterns

## Abstract

The present study aimed to (1) examine the changes in sleep habits and dietary intake among school-aged children after the school re-opening from a 3-month closure (without school lunch) due to the COVID-19 pandemic, and (2) examine whether the changes differ between those with different temporal patterns of sleep and eating during school closure, namely, ‘Very early’, ‘Early’, ‘Late’ and ‘Very late’. The latter patterns were characterised by later timings of wake up, breakfast and lunch. Questionnaires were answered twice by 4084 children (aged 8–15 years), themselves and/or their parents: first in June 2020 (for assessing lifestyle during school closure) and second, from July 2020 to February 2021 (for assessing lifestyle after school opening). After school re-opening, the participants’ wake-up time became an hour earlier (95 % CI 1⋅0, 1⋅1) and sleep duration got 0⋅94 h shorter (95 % CI 0⋅91, 0⋅97) than during school closure. An increase in dietary intake was observed for thiamine, vitamin B6, potassium, fruits and dairy products, and a decrease was observed for sugars (as foods) and confectioneries and sweetened beverages, despite small effect sizes (Cohen's *d*: 0⋅20–0⋅30). Significant changes in wake-up time, sleep duration and sweetened beverage intake were observed among children with the latter temporal patterns. Thus, children wake up earlier and sleep for shorter durations after school re-opening than during school closure; however, changes in dietary intake were generally insignificant. Dietary intake among school-aged children in Japan during school closure (without school lunch) might not be worse than that during school days with universal school lunch.

## Introduction

The coronavirus disease 2019 (COVID-19) pandemic has caused many schools across the world (including Japan) to close to avoid further spread of the highly contagious severe acute respiratory syndrome coronavirus 2 (SARS-CoV-2) virus^(1)^. According to the Ministry of Education, Culture, Sports, Science and Technology in Japan, by March 16, 2020, 99⋅0 % of all primary and secondary schools in the country were closed to avoid the further spread of the SARS-CoV-2 virus^(2)^. Studies have found that school schedule is one of the significant factors related to the lifestyle of school-aged children, such as sleep habits^(3,4)^ and dietary intake^(5)^. Thus, long-term school closure may affect sleep and dietary patterns and habits among school-aged children, which can thus, be detrimental to their health in the long run.

Previous studies examined changes in lifestyle behaviours among children during school closures or lockdowns^(6–12)^. Some studies, in particular, have reported changes in sleep habits^(6,7)^, dietary habits^(7–12)^ and diet quality among children in Canada^(6)^, China^(12)^, Italy^(8,11)^, Spain^(7)^, Greece^(10)^, five European and South American countries^(9)^. However, only a few studies have examined how children's lifestyles changed after school closures ended post the recent pandemic. In Japan, most primary and secondary schools were closed for 3 months due to the COVID-19 pandemic, which was more than the standard 1-month annual holiday that is present in schools in Japan. For example, a report mentioned that more than 80 % of primary-school-aged participants had a summer holiday of 40 d or less in 2016^(13)^. Therefore, lifestyle behaviour developed during school closures due to the pandemic could affect behaviour among school-aged children after school re-opening.

Furthermore, previous studies primarily assessed dietary variables before and during school closure using simple and/or non-validated questions^(8–10,12,14)^. Moreover, many previous studies assessed sleep and dietary habits before and during school closure at the same survey point^(6–10,12)^. Namely, participants answered questionnaires for assessing habits before school closure and those during school closure at the same time. However, only a limited number of longitudinal studies have quantitatively assessed dietary intake to examine the changes related to the COVID-19 pandemic^(11,15–17)^.

School-aged children might have experienced unusual daily lives during school closure, including education, sleep and diet. In Japan, the availability and content of educational assignments at home varied, depending on schools and municipalities. For example, while 100 % of primary schools and secondary schools provided assignment on textbooks and other paper materials, only 8 % of primary schools and 10 % of secondary schools provided interactive online programmes^(18)^. During daytime, children were stayed at home by themselves or with parents except for those who aged primary-school and registered as a user of after-school centres (i.e. a place for elementary school children whose parents are not at home to stay after school)^(19)^. A previous study in Japan showed that working mothers with primary school-age children were more likely to work from home, unlike fathers with primary school-age children and parents with secondary school-age children^(20)^. Thus, it is highly likely that most children did not have social obligations requiring them to get up early during school closure. In addition, children did not have school lunches during school closure. In the usual school year, the same lunch menu is provided to all children of a school under the Japanese school lunch programme. The nutrient content of school lunches is regulated by the Standards for the School Lunch Program to be nutritionally adequate. Thus, the absence of a school schedule and school lunch during school closure might alter lifestyle behaviours and dietary intakes among children.

In a previously conducted study of school-aged children in Japan, we identified four distinctive temporal patterns of sleep and eating during school closure due to the COVID-19 pandemic^(21)^. Unfavourable lifestyle behaviour and dietary intake were found among children with the latter temporal patterns characterised by delayed wake-up time and consuming breakfast. However, changes in sleep habits and dietary intake among school-aged children in Japan, post-school re-opening, have not been investigated yet. Furthermore, it is also unknown whether these changes are related to the temporal patterns of sleep and eating during school closures. Thus, the present study's aims were (1) to examine the changes in sleep habits and dietary intake from the 3-month school closure (without school lunch) due to the COVID-19 pandemic after school re-opening among school-aged children and (2) to examine whether the changes differ between those with different temporal patterns of sleep and eating during school closure based on a latent class analysis (LCA).

## Methods

### Study design and participants

The study design and participants have been reported in detail elsewhere^(21)^. The target population of this study included school-aged children (third to sixth graders of primary schools and first to third graders of secondary schools) aged 8–15 years. Participating schools, sports club and research collaborators were recruited via websites, social media and direct e-mails to previous collaborators. Students (*n* 11 958) from forty-three elementary and secondary schools, one sports club, and acquaintances of four research collaborators were then recruited. Questionnaires were distributed among the participants; the aim and procedure of the study were elucidated using a document attached to the questionnaires. Participants who answered and submitted the questionnaires were considered to agree to participate in the survey unless they indicated disagreement against using their data. This study was conducted in accordance with the guidelines of the Declaration of Helsinki. All procedures involving human participants were approved by the Ethics Committee of the University of Tokyo, Faculty of Medicine (approval no. 2020056NI, May 25, 2020).

All participants attended the schools that were closed from the first week of March 2020 to the last week of May or the first week of June 2020. The first survey was conducted immediately after the schools re-opened in June 2020 to assess the lifestyles of the participants during school closures. Participants were asked to recall and describe their lifestyle behaviour and dietary intake during the previous month, that is, during school closure. To assess lifestyle after school opening, the second survey was conducted at least 1 month after school re-opening to ensure the provision of school lunches on a regular basis. The timing of the second survey varied, depending on the school schedule and the new wave of infection. Two schools were surveyed in July and August 2020, thirty-seven schools, one sports club and four research collaborators in November to December 2020, and three other schools in February 2021. One school declined to participate in the second survey due to the school schedule. All schools attended by participants provide school lunches planned by dietitians in the usual school years. At least 1 month before the second survey, the supply of school lunches was restarted in the usual style.

### Measurements

In both surveys, participants were instructed to answer a questionnaire about lifestyle behaviours and dietary intake. The details of the questionnaires have been described previously^(21)^. The questionnaire for lifestyle behaviours during school closure included questions on clock time for waking up and going to bed and frequency and clock time for eating breakfast, lunch, dinner and a late-night snack. The questionnaire for lifestyle behaviours after school re-opening included questions on the clock time for waking up and going to bed. Responses for questions on the clock times for waking up and going to bed were answered in units of an hour and minutes (hh:mm). It was not asked whether the children wake up on their own or are woken up (by the alarm clock or the parents). Sleep duration was calculated using wake-up time and bedtime. The midpoint of sleep was calculated as the midpoint between bedtime and wake-up time as an indicator of chronotype, considering the association with sleep habits and chronotype^(22,23)^. Dietary intake during school closure and after re-opening was assessed using a brief-type self-administered diet history questionnaire for children and adolescents (BDHQ15y)^(24,25)^. It was filled by participating children themselves, one of the family members of the participating children (who were mainly responsible for meal preparation), or children and their family members together. The BDHQ15y is a four-page fixed-portion questionnaire concerning the frequency of consumption of selected food items commonly consumed in Japan, general dietary behaviours and usual cooking methods during the previous month. The BDHQ15y was developed based on the 16-page comprehensive^(26)^ and 4-page brief versions^(27,28)^ of a validated self-administered diet history questionnaire for Japanese adults. Estimates of daily intakes of foods (90 food items in total), energy and selected nutrients were calculated using an *ad hoc* computer algorithm for the BDHQ15y based on the Standard Tables of Food Composition in Japan^(29)^ and sex-specific fixed portion size. The BDHQ15y was validated for selected nutrients, including protein, fatty acids and carotenoids, using biomarkers (erythrocyte fatty acid and serum carotenoid levels) as a gold standard^(24,25)^. Nutrient and food intakes were adjusted by density methods and then expressed as % energy (for macronutrients) or unit per 4184 kJ (1000 kcal) to minimised the effect of reporting error of energy^(28)^.

Details about the date of birth, sex, body weight and height of the participants were self-reported as part of the BDHQ15y. Body mass index (BMI) during school closure was calculated as weight (kg) divided by the square of height (m). Weight status was defined by using the international age- and sex-specific cut-off points for thinness^(30)^ and overweight for children^(31,32)^. These cut-offs were derived from regression techniques applied to a large international sample. Children with BMI values that corresponded to an adult BMI <18⋅5, 18⋅5–24⋅9 and ≥ 25 kg/m^2^ were classified as underweight, normal weight and overweight, respectively.

Living status and sibling status were determined based on the answers to the questionnaire on lifestyle behaviours. Living status was categorised into two groups: (1) living with both parents and (2) living with a single parent and/or other relatives. Sibling status was categorised into two groups: (1) having at least one sibling at or under primary school age or (2) not having any siblings at or under primary school age.

Information on household income of the previous year (Japanese yen/year) and maternal education level was collected using the questionnaire in the second survey. For household income, sixteen categories were available, which were grouped into approximate tertiles for analysis: low (<5 million Japanese yen), middle (5–<8 million Japanese yen) and high (≥8 million Japanese yen). Maternal education level was categorised into three levels: low (12 years or less), middle (13–15 years) and high (16 years or more).

### Analysed participants

**Fig. 1.**
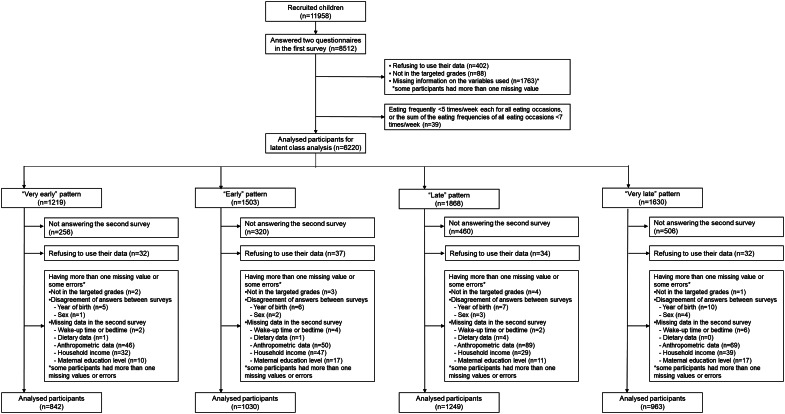
Flowchart of study participants’ selection.

Among 11 958 recruited children, 8512 (71 %) responded to the questionnaires of the first survey in June 2020. Out of the 8512 participants, 6220 children with interested variables were categorised into four classes by LCA^(21)^ (Fig. 1). Among 6220 children, those who did not respond to the second survey (*n* 1542) and those who refused to use their data (*n* 135) were excluded from the present analysis. Furthermore, we excluded participants who had one or more missing information or some errors in the variables used in the present analysis (*n* 459; *n* 10 for not in the targeted grades; *n* 28 for disagreement of the self-reported year of birth between surveys; *n* 10 for disagreement of self-reported sex between surveys; *n* 14 for missing wake-up time or bedtime; *n* 6 for missing dietary data; *n* 254 for missing anthropometric data; *n* 147 for household income; *n* 55 for maternal education level; some participants had more than one missing value or error). Thus, 4084 participants were included in the present study (34 % of invited children in the first survey [4084/11 958]; 66 % of the participants in the previous study [4084/6220]).

### Temporal pattern of sleep and eating

Temporal patterns of sleep and eating were determined using LCA by using SAS statistical software (version 9.4; SAS Institute Inc., Cary, NC, USA) with the PLOC LCA procedure (version 1.3.2)^(33,34)^, as used in a prior study^(21)^. In brief, based on the self-reported clock time for waking up, going to bed and eating meals, thirty binary variables (1 = event, 2 = no-event), which indicated whether the event occurred in each time frame, were generated as input variables for LCA. Four classes were selected as an optimal number of classes considering model fit indices using the Bayesian information criterion, Akaike information criterion (AIC), adjusted AIC, entropy and interpretability of patterns. The first pattern was labelled ‘Very early’, as participants reported an earlier timing of waking up and breakfast than those in the other patterns. The second pattern was labelled ‘Early’, where participants woke up and ate breakfast 1 h later than those in the first pattern. The third pattern was labelled ‘Late’, as the participants in this pattern woke up and ate breakfast 1–2 h later than those in the first pattern. The fourth pattern, labelled ‘Very late’ included participants who woke up and consumed breakfast the most late. The following number of participants were included in each pattern in the present study: ‘Very early’ (*n* 842/1219, 69 % of the previous study), ‘Early’ (*n* 1030/1503, 68 %), ‘Late’ (*n* 1249/1868, 67 %) and ‘Very late’ (*n* 963/1630, 60 %) patterns.

### Statistical analyses

All statistical analyses were performed using the SAS 9.4 statistical software. All reported *P*-values were two-tailed, and a *P*-value < 0⋅05 was considered to be statistically significant. Data were presented as means and standard deviations (sds) for continuous variables and as numbers and percentages for categorical variables. In the adjusted model, data were presented as means and standard errors (ses) for continuous variables. The mean (sd) clock times for eating meals were calculated for participants who ate each type of meal ≥5 times per week.

The basic characteristics were then compared between the excluded participants (*n* 2136) and the analysed participants (*n* 4084) from 6220 eligible participants^(21)^. Unpaired *t* tests were conducted for continuous variables, and the χ^2^ test was used for categorical variables.

The basic characteristics were also compared between different temporal patterns of sleep and eating. Mean differences in continuous variables (i.e. age, height, weight and BMI) among LCA-derived classes were tested with linear regression models using the PROC GLM procedure. An ordinal scale (i.e. 1 = ‘Very early’, 2 = ‘Early’, 3 = ‘Late’ and 4 = ‘Very late’ pattern) according to circadian timing was assigned for each class and used as a continuous independent variable in the linear regression models. Differences in categorical variables were tested using the χ^2^ test.

Changes in the timings of waking up and going to bed, sleep duration, midpoint of sleep as well as dietary intake were tested using paired *t* tests. To estimate the effect size of school re-opening, Cohen's *d* was determined by dividing the mean difference between the two survey periods by the *pooled* standard deviation. Cohen's *d* of 0⋅2, 0⋅5 and 0⋅8 were considered as a ‘small’, ‘medium’ and ‘large’ effect size, respectively^(35,36)^. Given the large *sample size* of this study, even very small effect sizes could produce *significant P-value*s (<0⋅05). Due to the risk of artificial *P-value* deflation^(37)^, the results with *P* < 0⋅05 and Cohen's *d* ≥ 0⋅2 were interpreted as significant.

Differences in sleep habits and dietary intake during school closure between temporal patterns were tested with linear regression models using the PROC GLM procedure. The ordinal scale of sleeping and eating pattern was used as independent variable and variables regarding sleep habits and dietary intake as dependent variables. Similarly, differences in sleep habits and dietary intake after school re-opening between the temporal patterns were also tested. Moreover, linear regression models were used to test the differences in changes in these outcome variables among different temporal patterns. Partial eta-squared variables (*η*^2^*_p_*) were used to estimate the effect size. *η*^2^*_p_* = 0⋅01, 0⋅25 and 0⋅64 were considered as a ‘small’, ‘medium’ and ‘large’ effect size, respectively^(35,36)^. A multivariate-adjusted model analysis was also performed with age, sex, living status and sibling status as confounding variables.

To examine whether the changes differed between those with different temporal patterns of sleep and eating during school closure, repeated measures of ANOVA were conducted for each dependent variable (i.e. wake-up time, bedtime, sleep duration, midpoint of sleep, nutrient intake and food intake). In the crude model, time (during school closure (ref) *v*. after school re-opening), class (four temporal patterns; ref = ‘Very early’ patterns) and time × class were included as independent variables. Sex, age, weight status, living status, sibling status, household income level and maternal education level were included in the adjusted model.

### Sensitivity analysis

Sensitivity analysis was performed only on participants with a more plausible reported energy intake (*n* 3939). Participants were excluded if their energy intake estimated using the BDHQ15y was <0⋅5 times the estimated energy requirement (EER) for children with the lowest physical activity level or ≥1⋅5 times the EER for those with the highest physical activity level.

Another sensitivity analysis was performed on the participants who answered the questionnaires in the second survey from November to February 2020 (*n* 3980). Those who responded to the second survey questionnaire in the other period were excluded from the analysis to regulate the seasonal effect of sleep habits and dietary intake.

## Results

Participants excluded from the present analysis were older, being boys, and living with a single parent and/or other relative (Supplementary Table S1). Furthermore, those categorised as ‘Very late’ or ‘Late’ tended to be excluded from the present analysis.

Table 1 shows the characteristics of the participants according to the LCA-derived temporal patterns of sleeping and eating classes. Participants with the latter pattern tended to be older and being girl. The proportion of participants living with a single parent and/or other relatives and those having sibling(s) aged at an equivalent of or under primary school was higher in the latter patterns. The prevalence of low-income level and low maternal education level was higher in the ‘Very late’ pattern than in the other patterns. The clock times for waking up, going to bed and eating meals during school closure were earlier for children with earlier patterns than those with the latter patterns (Supplementary Table S2).

**Table 1.** Baseline characteristics of 4084 school-aged children (third to sixth grade of primary school and first to third grade of secondary school) according to latent class analysis-derived sleeping and temporal eating pattern classes

	‘Very early’ pattern				‘Early’ pattern				‘Late’ pattern				‘Very late’ pattern				P*
	*n* 842				*n* 1030				*n* 1249				*n* 963				
	*N*	(%)	Mean	sd	*N*	(%)	Mean	sd	*N*	(%)	Mean	sd	*N*	(%)	Mean	sd	
Age during school closure (y)			10⋅6	1⋅8			10⋅7	1⋅8			10⋅8	1⋅8			11⋅4	1⋅9	<0⋅001
Height during school closure (cm)			142⋅6	11⋅7			143⋅5	11⋅8			143⋅8	12⋅1			147⋅2	12⋅4	<0⋅001
Weight during school closure (kg)			36⋅7	9⋅7			37⋅2	10⋅0			37⋅8	10⋅9			40⋅4	10⋅8	<0⋅001
Body mass index (kg/m^2^)			17⋅8	2⋅7			17⋅8	2⋅8			18⋅0	2⋅9			18⋅3	2⋅8	<0⋅001
Weight status																	0⋅65
Underweight	84	(10)			114	(11)			138	(11)			89	(9)			
Normal weight	643	(76)			777	(75)			938	(75)			725	(75)			
Overweight	115	(14)			139	(13)			173	(14)			149	(15)			
Sex (boys)	472	(56)			515	(50)			603	(48)			425	(44)			<0⋅001
Grade																	<0⋅001
Primary school	629	(75)			757	(73)			901	(72)			552	(57)			
Secondary school	213	(25)			273	(27)			348	(28)			411	(43)			
Family structure																	0⋅001
Living with both parents	756	(90)			924	(90)			1130	(90)			823	(85)			
Living with single parent and/or other relatives^a^	86	(10)			106	(10)			119	(10)			140	(15)			
Sibling status: having sibling(s) aged at or under primary-school-age																	<0⋅001
Yes	359	(43)			491	(48)			589	(47)			562	(58)			
No	483	(57)			539	(52)			660	(53)			401	(42)			
Household income																	<0⋅001
Low (5 million Japanese yen)	249	(30)			299	(29)			368	(29)			373	(39)			
Middle (5–<8 million Japanese yen)	324	(38)			399	(39)			492	(39)			340	(35)			
High (≤8 million Japanese yen)	269	(32)			332	(32)			389	(31)			250	(26)			
Maternal education level																	<0⋅001
Low (≤12 years)	171	(20)			206	(20)			260	(21)			300	(31)			
Middle (13–15 years)	376	(45)			447	(43)			588	(47)			439	(46)			
High (≥16 years)	295	(35)			377	(37)			401	(32)			224	(23)			

a Age- and sex-specific body mass index (BMI) cut-offs by the International Obesity Task Force (refs. 30,31) were used. Underweight was defined according to the cut-offs corresponding to BMI for adults of <18⋅5 kg/m^2^ (ref. 29). Normal weight was defined according to the cut-offs corresponding to an adult BMI of ≤18⋅5 and >25 kg/m^2^ (refs. 30,31). Overweight was defined according to the cut-offs corresponding to an adult BMI of ≥25 kg/m^2^ (ref. 31).

* *P*-values represent *P* for trend by sleeping and eating pattern for continuous variables, and for categorical variables the *P*-values were tested by χ^2^ test. Trend of association was examined using a linear regression model with the ordinal scale of sleeping and eating pattern (1 = ‘Very early’ pattern, 2 = ‘Early’ pattern, 3 = ‘Late’ pattern and 4 = ‘Very late’ pattern) as a continuous variable.

After the school re-opened, the wake-up time was recorded to be 1⋅0 h earlier (*P* < 0⋅001) and sleep duration was 0⋅94 h shorter (*P* < 0⋅001), midpoint of sleep was 0⋅5 h earlier (*P* < 0⋅001) than during school closure for the whole sample (Table 2). An increase in intake was observed for thiamine, vitamin B6, potassium, fruits and dairy products, and a decrease in intake was observed for sugars, confectioneries and sweetened beverages (Cohen's *d* ≥ 0⋅02). There were other nutrient and food groups, the intake of which also increased or decreased with statistical significance, but the mean difference was very small (Cohen's *d* < 0⋅02). The number of participants with zero intake decreased for pulses, fruits, fish and shellfish, and dairy products, and increased for sweetened beverages.

**Table 2.** Sleep habits and dietary intakes during school closure and after school re-opening among 4084 school-aged children (third to sixth grade of primary school and first to third grade of secondary school)

	During school closure			After school re-opening			Difference from school closure		
	0 intake children [*N* (%)]	Mean^a^	sd	0 intake children [*N* (%)]	Mean^a^	sd	Mean (95 % CI)	P*	Cohen's *d*^b^
Sleep habits									
Wake-up time (clock time; hh:mm)	–	7:35	1:06	–	6:32	0:29	−1⋅0 (−1⋅1, −1⋅0)§	<0⋅001	1⋅23
Bed time (clock time; hh:mm)	–	22:15	0:58	–	22:09	0:52	−0⋅11 (−0⋅13, −0⋅08)§	<0⋅001	0⋅12
Sleep duration (h)	–	9⋅3	0⋅9	–	8⋅4	0⋅8	−0⋅94 (−0⋅97, −0⋅91)	<0⋅001	1⋅04
Midpoint of sleep (clock time; hh:mm)		2:55	0:55		2:21	0:33	−0⋅58 (−0⋅60, −0⋅55)	<0⋅001	0⋅76
Nutrient intake									
Protein (% of energy)	–	14⋅0	2⋅2	–	14⋅3	2⋅2	0⋅31 (0⋅27, 0⋅36)	<0⋅001	0⋅14
Total fat (% of energy)	–	31⋅0	5⋅3	–	30⋅3	5⋅6	−0⋅70 (−0⋅78, −0⋅63)	<0⋅001	0⋅13
Saturated fatty acids (% of energy)	–	10⋅2	2⋅4	–	9⋅9	2⋅4	−0⋅30 (−0⋅35, −0⋅25)	<0⋅001	0⋅13
Carbohydrate (% of energy)	–	53⋅6	6⋅3	–	54⋅0	6⋅6	0⋅39 (0⋅31, 0⋅47)	0⋅0006	0⋅06
Dietary fibre (g/4184 kJ)	–	5⋅7	1⋅5	–	5⋅9	1⋅6	0⋅18 (0⋅14, 0⋅21)	<0⋅001	0⋅11
Vitamin A (μg retinol equivalents/4184 kJ)	–	315	173	–	346	181	31⋅0 (30⋅6, 31⋅4)	<0⋅001	0⋅18
Thiamine (mg/4184 kJ)	–	0⋅40	0⋅07	–	0⋅42	0⋅07	0⋅02 (0⋅01, 0⋅03)	<0⋅001	0⋅29
Riboflavine (mg/4184 kJ)	–	0⋅72	0⋅17	–	0⋅75	0⋅18	0⋅02 (0⋅01, 0⋅04)	<0⋅001	0⋅14
Niacin (mg/4184 kJ)	–	7⋅1	1⋅7	–	7⋅3	1⋅7	0⋅27 (0⋅23, 0⋅31)	<0⋅001	0⋅16
Vitamin B6 (mg/4184 kJ)	–	0⋅56	0⋅13	–	0⋅59	0⋅13	0⋅03 (0⋅02, 0⋅04)	<0⋅001	0⋅23
Vitamin B12 (μg/4184 kJ)	–	3⋅4	1⋅7	–	3⋅5	1⋅6	0⋅15 (0⋅11, 0⋅19)	<0⋅001	0⋅09
Folate (μg/4184 kJ)	–	161	53	–	169	56	8⋅5 (8⋅3, 8⋅7)	<0⋅001	0⋅16
Vitamin C (mg/4184 kJ)	–	53⋅9	22⋅2	–	57⋅9	23⋅8	4⋅0 (3⋅8, 4⋅1)	<0⋅001	0⋅17
Sodium (mg/4184 kJ)	–	1899	399	–	1843	406	−55⋅9 (−56⋅5, −55⋅2)	<0⋅001	0⋅14
Potassium (mg/4184 kJ)	–	1172	269	–	1234	285	61⋅7 (61⋅2, 62⋅2)	<0⋅001	0⋅22
Calcium (mg/4184 kJ)	–	325	105	–	342	109	17⋅5 (17⋅2, 17⋅8)	<0⋅001	0⋅16
Magnesium (mg/4184 kJ)	–	119	23	–	123	23	4⋅3 (4⋅1, 4⋅4)	<0⋅001	0⋅19
Iron (mg/4184 kJ)	–	3⋅8	0⋅9	–	3⋅9	0⋅9	0⋅05 (0⋅02, 0⋅08)	<0⋅001	0⋅06
Food intake (g/4184 kJ)									
Cereal	2 (0)	217	62	5 (0)	222	66	4⋅9 (4⋅7, 5⋅2)	<0⋅001	0⋅08
Sugars and confectioneries	0 (0)	53⋅1	27⋅7	1 (0)	43⋅7	26⋅0	−9⋅4 (−9⋅6, −9⋅3)	<0⋅001	0⋅35
Pulses	68 (2)	25⋅4	17⋅4	59 (1)	26⋅1	17⋅8	0⋅7 (0⋅6, 0⋅8)	0⋅007	0⋅04
Vegetables	3 (0)	108⋅8	57⋅5	4 (0)	116⋅3	62⋅4	7⋅5 (7⋅3, 7⋅7)	<0⋅001	0⋅12
Fruits	281 (7)	28⋅1	27⋅4	188 (4)	37⋅5	33⋅7	9⋅4 (9⋅2, 9⋅5)	<0⋅001	0⋅30
Fish and shellfish	27 (1)	27⋅7	15⋅6	22 (1)	28⋅7	15⋅6	1⋅08 (0⋅96, 1⋅20)	<0⋅001	0⋅07
Meat	3 (0)	39⋅4	16⋅7	6 (0)	40⋅3	17⋅0	0⋅95 (0⋅81, 1⋅08)	0⋅002	0⋅06
Dairy products	71 (2)	104⋅9	86⋅6	47 (1)	122⋅2	86⋅7	17⋅3 (17⋅0, 17⋅6)	<0⋅001	0⋅20
Sweetened beverages	842 (20)	42⋅1	68⋅9	1118 (26)	27⋅7	48⋅9	−14⋅4 (−14⋅6, −14⋅1)	<0⋅001	0⋅24

a Participants with zero intake were included in the calculation of means.

b *d* = 0⋅2, 0⋅5 and 0⋅8 were considered as a ‘small’, ‘medium’ and ‘large’ effect size.

* *P* for the paired *t* test. *P* < 0⋅05 and Cohen's *d* ≥ 0⋅2 was considered statistically significant.

**Fig. 2.**
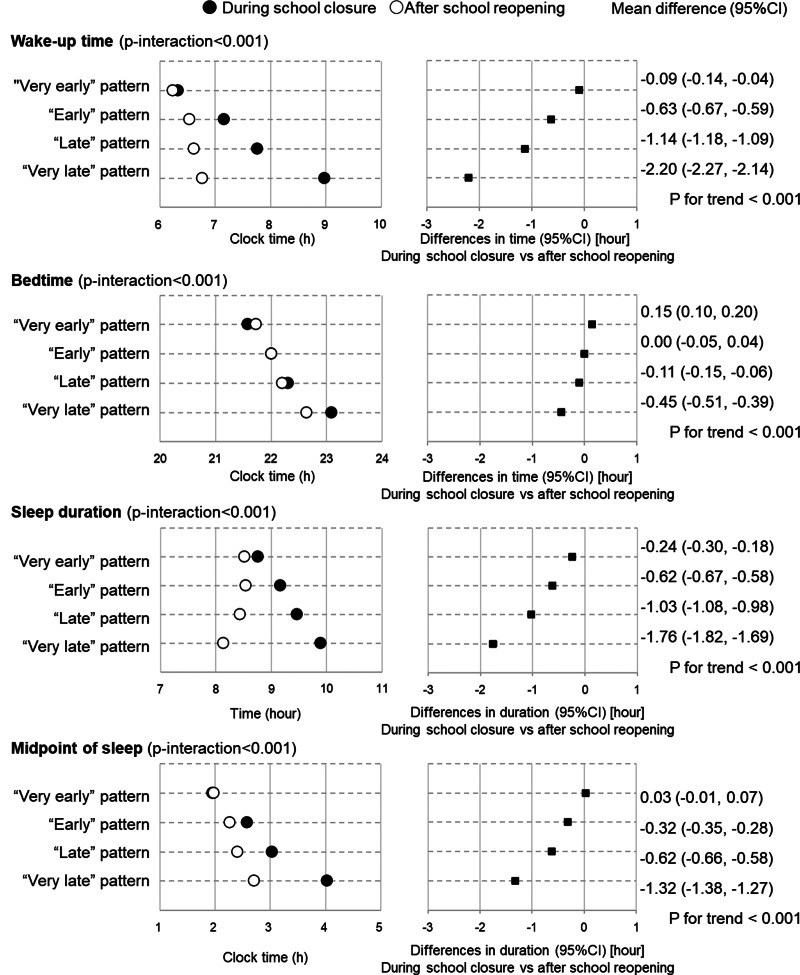
Change in sleep habits among 4084 school-aged children according to latent class analysis-derived sleeping and temporal eating pattern classes. Number of participants in each pattern are: ‘Very early’ pattern (*n* 842), ‘Early’ pattern (*n* 1030), ‘Late’ pattern (*n* 1249) and ‘Very late’ pattern (*n* 963). *P*-values represent *P* for trend by sleeping and eating pattern for continuous variables. Trend of association of difference was examined using a linear regression model with the ordinal scale of sleeping and eating patterns (1 = ‘Very early’ pattern, 2 = ‘Early’ pattern, 3 = ‘Late’ pattern and 4 = ‘Very late’ pattern) as a continuous variable. Closed circles present values during school closure and open circles represent presents after school re-opening.

After school re-opening, children with earlier patterns still had earlier clock times for wake-up (*P* < 0⋅001), going to bed (*P* < 0⋅001) and midpoint of sleep (*P* < 0⋅001) (Supplementary Table S3 and Fig. 2). Moreover, children with ‘Very late’ patterns had the shortest sleep duration after school re-opening, whereas they had a longer sleep duration than those with patterns during school closure. In both survey periods, participants with the earlier pattern had higher intakes of protein, some vitamins and minerals, pulses, vegetables, fruits, fish and shellfish, and dairy products, and lower intakes of sugar and confectionaries and sweetened beverages, but the effect sizes were very small (*η*^2^*_p_* < 0⋅04). These associations were similar after adjusting for age, sex, living status and presence of siblings (Supplementary Table S4).

**Fig. 3.**
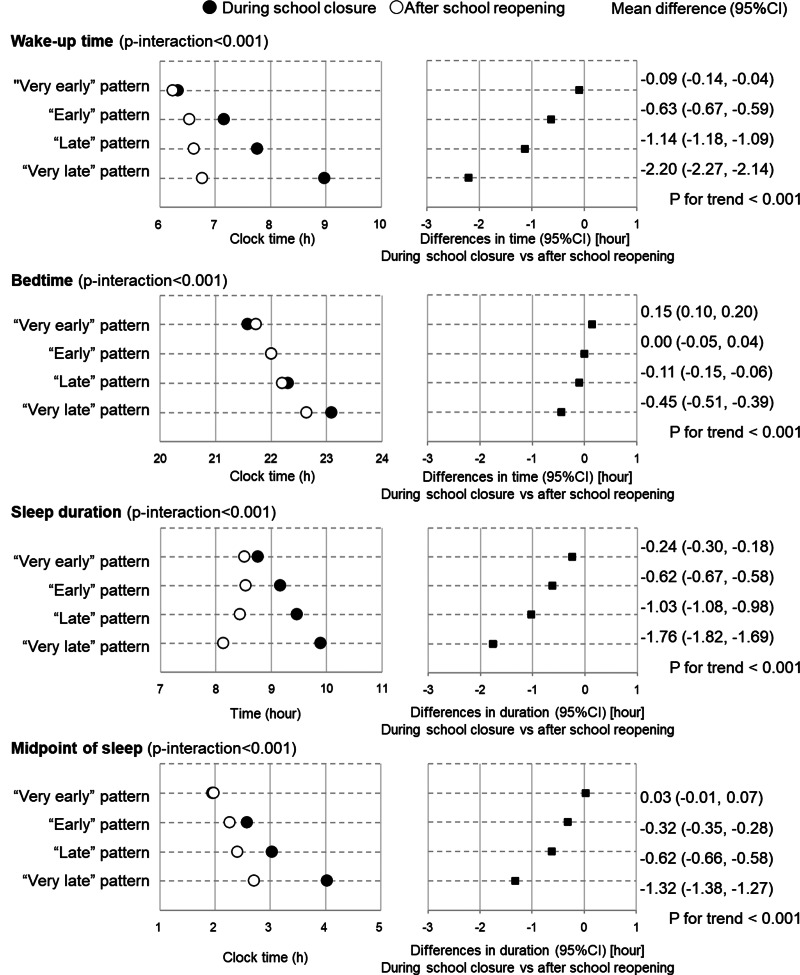
Change in selected nutrient intakes among 4084 school-aged children according to latent class analysis-derived sleeping and temporal eating pattern classes. Number of participants in each pattern are: ‘Very early’ pattern (*n* 842), ‘Early’ pattern (*n* 1030), ‘Late’ pattern (*n* 1249) and ‘Very late’ pattern (*n* 963). *P*-values represent *P* for trend by sleeping and eating pattern for continuous variables. Trend of association of difference was examined using a linear regression model with the ordinal scale of sleeping and eating patterns (1 = ‘Very early’ pattern, 2 = ‘Early’ pattern, 3 = ‘Late’ pattern and 4 = ‘Very late’ pattern) as a continuous variable. Closed circles present values during school closure and open circles represent presents after school re-opening.

**Fig. 4.**
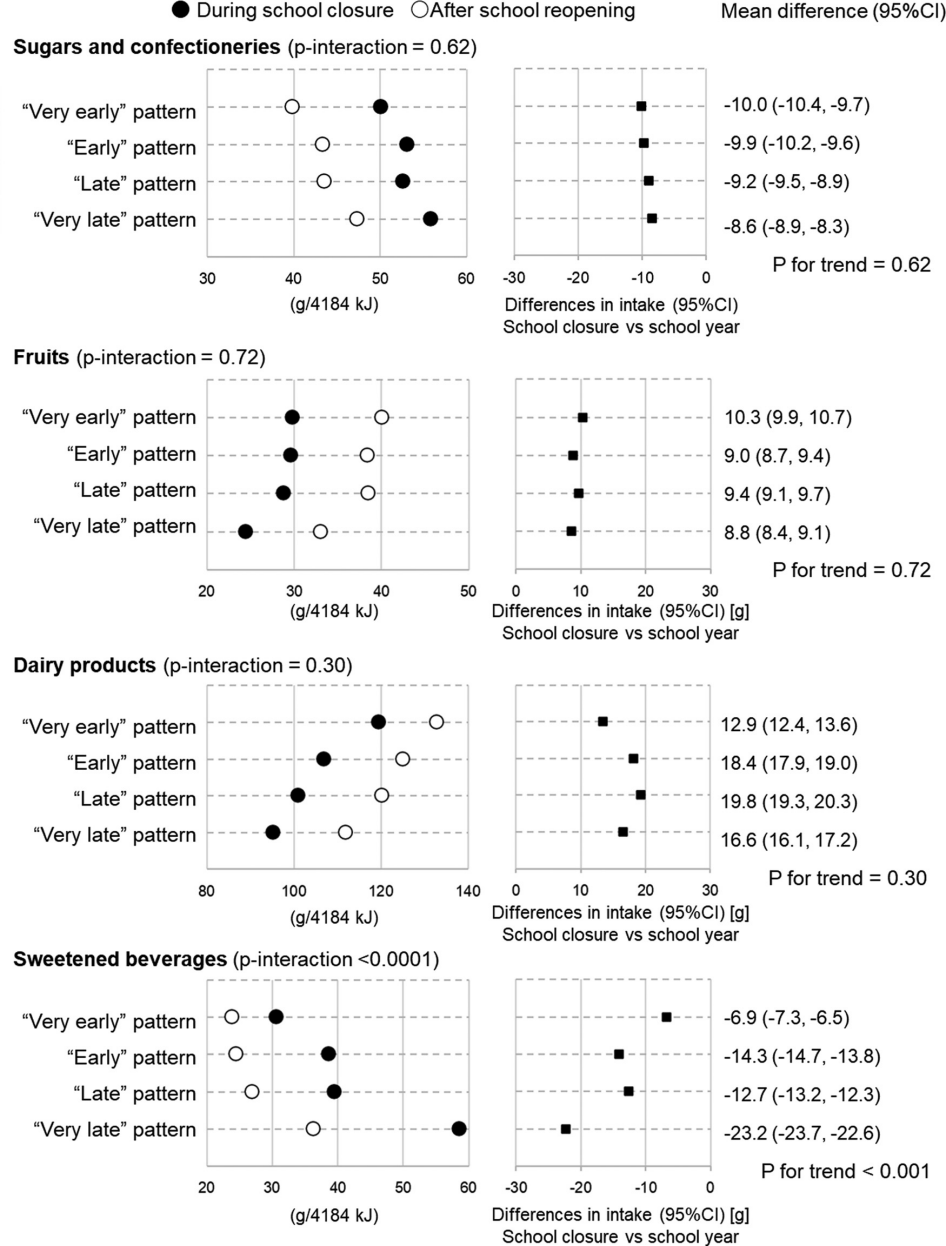
Change in selected food intakes among 4084 school-aged children according to latent class analysis-derived sleeping and temporal eating pattern classes. Number of participants in each pattern are: ‘Very early’ pattern (*n* 842), ‘Early’ pattern (*n* 1030), ‘Late’ pattern (*n* 1249) and ‘Very late’ pattern (*n* 963). *P*-values represent *P* for trend by sleeping and eating pattern for continuous variables. Trend of association of difference was examined using a linear regression model with the ordinal scale of sleeping and eating patterns (1 = ‘Very early’ pattern, 2 = ‘Early’ pattern, 3 = ‘Late’ pattern and 4 = ‘Very late’ pattern) as a continuous variable. Closed circles present values during school closure and open circles represent presents after school re-opening.

Larger changes in wake-up time, bedtime and sleep duration were observed among children with the latter temporal pattern (Fig. 2; Supplementary Tables S5 and S6). Figs. 3 and 4 and Supplementary Table S4 show the changes in dietary intake according to the temporal pattern classes. The figures only show results for nutrients and foods with differences of *P* < 0⋅05, and Cohen's *d* > 0⋅02 in the paired *t* test for the whole sample, namely, thiamine, vitamin B6, potassium, sugar and confectionaries, fruits, dairy products and sweetened beverages. Greater changes in the intake of sweetened beverages were observed in children with the latter temporal patterns. Except for meat, no significant time × class interaction were observed for the other nutrients and food items (Supplementary Table S6). These associations were similar after adjusting for age, sex, living status and presence of siblings (Supplementary Table S7).

Sensitivity analyses limiting analysed participants with plausible reported energy intake (*n* 3939) and those who responded to the second survey from November 2020 to February 2021 (*n* 3980) did not considerably alter the abovementioned findings (Supplementary Tables S8 and S9).

## Discussion

To our knowledge, this is the first study to examine changes in sleep and dietary intake among school-aged children during and after school closure due to the COVID-19 pandemic. The primary finding of the present study was that children got up earlier and slept for a shorter duration after the re-opening of school than they did during school closure. Moreover, significant changes in sleeping habits were observed in children who had a later timing of sleeping and eating during school closure. However, differences in dietary intake were generally insignificant. Furthermore, changes in dietary intake did not differ among children with different temporal pattern classes, except for sweetened beverages.

The analysis found that the participants woke up earlier after school re-opening than during school closure. This is consistent with several previous studies reporting that children had longer sleep durations during the COVID-19 outbreak than before the outbreak^(6,7)^. Other studies have shown that school time is associated with shorter sleep durations among children and adolescents^(3,4)^. Thus, going to school had a significant effect on sleeping habits among school-aged children, especially for the clock time for waking up.

A significant difference in wake-up time and sleep duration was noted in children with a later timing of sleep and eating during school closure. Similar to the findings of this study, a previous study found considerable delays in wake-up time and bedtime among adults with ‘evening’ chronotype during lockdown due to COVID-19 than before, compared with ‘intermediate’ and ‘morning’ chronotypes^(38)^. Furthermore, a more frequent social jet lag was observed among children with evening chronotype^(39)^. It is possible that children with ‘Very late’ patterns had an ‘evening’ chronotype. Moreover, after the school re-opened, children with ‘Very late’ patterns had shorter sleep durations than those with other patterns. It was also suggested that the starting times of schools in Japan were too early for evening chronotype children to have enough sleep duration.

It was found that children had more favourable dietary intakes after school re-opening than during school closure, for certain nutrients and food items. These results are partly consistent with previous studies showing that children had unfavourable dietary habits during school closure or lockdown, such as eating more snacks^(10,11)^, sugary drinks^(10,11)^ and a lower frequency of eating fruits^(12)^. Furthermore, the results also consistent with a previous study showing more favourable dietary intakes during weekdays with school lunches than weekend among school-aged children^(5)^. Thus, after the re-opening of school, having daily school lunches might improve the dietary intake among the participants. However, changes observed in this study were relatively small. Although the exact reason for the relatively small change is unknown, it may be caused by the changes in attitudes towards health and dietary intake due to the pandemic situation. Prior studies have found an improvement in dietary intake during school closures or lockdowns^(7,14,16,40)^. It is likely that some parents of participants in this study tried to prepare for their children healthy diets during school closures because of the pandemic. These possible changes in attitude to health would lead to small differences in dietary intake during and after school closure. Thus, it can be concluded that the dietary intake among school-aged children in Japan might not be worse during school closure due to the COVID-19 pandemic than before the school closure.

The present study had several limitations. The analysed participants were not nationally representative but a convenient sample. The study area was limited to only fourteen of the forty-seven prefectures in Japan as participants were recruited via voluntary schools or research collaborators but were not randomly selected. Furthermore, the analysed participants possibly had different characteristics compared to the non-participants and those excluded from the analysis. In fact, the majority of the analysed participants were older and male. Moreover, the retention rate among children with ‘Very late’ and ‘Late’ patterns was lower than those in the earlier patterns. Thus, it can be assumed that participants with relatively positive and favourable lifestyles tended to be included in the present analysis. Thus, these selection biases could make changes between survey periods and between temporal pattern classes, smaller. Second, the survey period may have affected participants’ answers. During the first survey, questionnaires were distributed after the schools re-opened. Thus, participants’ lifestyle behaviours during school closures were not fully reflected in the answers. However, the four identified temporal patterns had markedly different characteristics, especially the timing of waking up and having breakfast. Thus, the effect of recall bias in the first survey may be low. In the second survey, the survey period differed by school. However, sensitivity analysis based on the period of the second survey showed little difference in the study results. Thus, the effect of the difference in survey period on the results may be low. Third, the variables used for the analysis were assessed using a self-administered questionnaire whose validity was unknown or may be insufficient. Low correlation coefficients were found for all examined nutrients between intakes assessed by the BDHQ15y and the biomarkers^(24,25)^. Insufficient accuracy of the BDHQ15y could result in a small effect size for changes in dietary intake between the survey period and temporal eating patterns. Furthermore, non-validated questions were used to assess the sleep habits. A previous systematic review found a high correlation between self-reported sleep time and accelerometer use among children^(41)^. Thus, the observed changes in self-reported sleep habits are acceptable. However, the questionnaire has the potential for measurement errors resulting from recall bias and social desirability bias^(42)^. Parents of participating children possibly reported later wake-up times and earlier bedtimes than when their children actually went to bed and woke up^(43)^. These possible reporting biases could diminish the changes in sleep habits between the survey period and the temporal dietary patterns. Nevertheless, we observed distinctive differences between the survey period and temporal patterns. Thus, the effect of possible measurement errors may be small in the analysis of sleep habits.

In conclusion, among the participating school-aged children in Japan, the clock time for waking up occurred earlier, and sleep duration decreased after the re-opening of school after a school closure due to the COVID-19 pandemic. Larger and significant changes in these variables were associated with the later timing of waking up and breakfast consumption during school closure. However, changes in dietary intake were generally insignificant. It was found that dietary intake among school-aged children in Japan during school closures might not be worse than that during school days with the provision of universal school lunches. These findings will contribute to the development of future strategies to promote healthy sleep and dietary habits during long school closures due to emergencies like the pandemic and natural disasters or even during the re-opening of school after summer holidays.

## Supplementary material

For supplementary material accompanying this paper visit https://doi.org/10.1017/jns.2022.116. click here to view supplementary material
